# Supplementary material for: The topology of the reaction stereo-dynamics in chemi-ionizations
Source: Commun Chem. 2023 Feb 13;6:30. doi: 10.1038/s42004-023-00830-8 (PMC9925729; doi:10.1038/s42004-023-00830-8)
Supplement: Supplementary file 2 — Supplementary Information [file 42004_2023_830_MOESM2_ESM.pdf]

SUPPLEMENTARY INFORMATION

The topology of the reaction stereo-dynamics in chemi-ionizations

Stefano Falcinelli,[1] Franco Vecchiocattivi[1] and Fernando Pirani,[1,2]*

[1]Department of Civil and Environmental Engineering, University of Perugia, Via G. Duranti 93, 06125 Perugia, Italy.

[2]Department of Chemistry, Biology and Biotechnologies, University of Perugia, Via Elce di Sotto 8, 06123 Perugia, Italy.

Supplementary Methods

Additional details on the Optical Potential formulation

The theoretical treatment, presented in this paper and stimulated by the PIES and mass spectrometric measurements in our laboratory, is able to rationalize in a unifying picture all the observables quoted above, probing different features of the so called Optical Potential Model:[1-3]

$$W = V_t - \frac{i}{2}\Gamma \qquad (1)$$

Whose, the real part, V_t, controls the dynamical evolution of the reagents during each collision event, while the imaginary component, Γ, defines the reactivity, that is the probability of passage from neutral entrance to ionic exit channels for each configuration of the collision

complex. This is quantified by the lifetime of the autoionizing system at the separation distance R expressed by the eq. (2):

$$\tau(R) = \frac{\hbar}{\Gamma(R)} \quad (2)$$

The ionization probability at any distance R is given by the following eq. (3):

$$P(R)dR = \frac{\Gamma(R)dR}{\hbar g \sqrt{1 - \frac{V(R)}{E} - \frac{b^2}{R^2}}} \quad (3)$$

where g represents the relative asymptotic velocity, E the collision energy and b the impact parameter.

During a complete collision, the probability that the system survives (i.e. does not give rise to ionization) from infinite distance to the collision turning point R_c is:

$$F_{R_c,\infty}(b,g) = exp\left[-\int_{R_c}^{\infty} P(R)dR\right] = exp\left[-\int_{R_c}^{\infty} \frac{\Gamma(R)dR}{\hbar g \sqrt{1 - \frac{V(R)}{E} - \frac{b^2}{R^2}}}\right] \quad (4)$$

Finally, the total ionization cross section is given by the eq. (5) below.

$$\sigma_{tot}(g) = 2\pi \int_0^{\infty} P(b)bdb = 2\pi \int_0^{\infty}\left[1 - F_{R_c,\infty}^2(b,g)\right]bdb \quad (5)$$

The adopted methodology is able to reproduce all experimental data available for the Ne^*-Ar, N_2 and NH_3 systems obtained from our and other laboratories, including total and partial ionization cross sections and branching ratios between selected channels. Therefore, the proposed theoretical approach, which also includes within the same framework exchange and radiative mechanisms proposed in the past[3], is general and can be used to describe in a state-to-state condition the reactivity of all chemi-ionization reactions, including those involving molecules.[4,5]

For the real component V_t of the Optical Potential of eq. (1), the adopted formulation providing for the entrance channels the dependence of the isotropic interaction on the reagent separation distance R, leads to this expression:[6]

$$V_t(R) = S(R)\, V(R)^{neut.-neut.} + \big(1 - S(R)\big)\, V(R)^{ion-neut.} \tag{6}$$

where $V(R)^{neut.-neut.}$ and $V(R)^{ion-neut.}$ are represented by the Improved Lennard Jones (ILJ) function, whose general form is:[6]

$$V_{ILJ}(R) = \varepsilon \left[\frac{m}{n(R)-m}\left(\frac{R_m}{R}\right)^{n(R)} - \frac{n(R)}{n(R)-m}\left(\frac{R_m}{R}\right)^{m}\right] \tag{7}$$

with

$$n(R) = \beta + 4\left(\frac{R}{R_m}\right)^2 \tag{8}$$

Here ε is the potential well depth and R_m is its location, while $n(R)$ defines the hardness of the repulsive wall and the radial modulation of the attraction. The switching function $S(R)$, that accounts for the transition from the neutral-neutral to the ion-neutral representation of the interaction, as previously[5] it has been defined as

$$S(R) = \frac{1}{1 + e^{\left(\frac{R_o - R}{d}\right)}} \tag{9}$$

Here, R_o is the distance where the two combined limiting potential forms have the same weight, while *d* describes how fast the transition occurs.

The *R* dependence of the isotropic component of the interaction in the exit channel is represented again by an ILJ function. Basic aspects of the potential formulation for atom-atom reactions are discussed in a previous article[7] where it is shown that the parameter values were obtained from a semi-empirical method founded on the ample phenomenology of the interactions of open-shell “P” atoms (particularly halogen atoms). They have been investigated in detail with scattering experiments, performed with state selected atomic beams and analysed with a proper theoretical treatment.[8-10] Details on the extension of the

formulation to atom-molecule reactions, including the angular dependence of both terms of the optical potential *W*, are given in ref. 7 and references therein.

Additional Details on the experimental determinations

The experiments, performed under single collision condition with the molecular beam (MB) technique, allowed the measure of total and partial ionization cross sections[11-15], branching ratios[16,17] and Penning Ionization Electron Spectra (PIES)[15,18,19]

A scheme of the MB machine operating in our laboratory is shown in Supplementary Fig. 1. A primary beam of $Ne^*(^3P_J$, with J=2,0) atoms, emerging from an electron bombardment effusive or supersonic seeded source, crosses at right angles the secondary beam of target particles (Ar, N_2 or NH_3). PIESs have been measured exploiting a hemispherical electron energy analyzer located above the beam crossing volume, while total, partial cross sections and branching ratios have been determined by mass spectrometry using a quadrupole mas filter placed below the beam scattering center. It consists of three vacuum chambers: the first one contains the rare gas beam source, while in the second chamber the rare gas atoms are electronically excited and pulsed by a slotted disk; in the third chamber the metastable atoms cross the target molecules of a secondary effusive beam. In this chamber the metastable atoms are monitored, while product ions and emitted electrons are detected, after mass analysis, for the ions, and energy selection, for the electrons. The neon beam can be produced by two sources that can be used alternately. The first one is a standard effusive source at room temperature while the second one is a supersonic device that can be heated to different temperatures. In both cases the metastable atoms are produced by electron bombardment at about 150 eV, that is expected to yield $Ne(^3P_2)$ and $Ne(_3P_0)$ in a population close to the statistical 5:1 ratio.[4] The metastable atom velocity can be analyzed by a time-of-flight (TOF) technique. The resolution of our electron spectrometer is of about 45 meV at a transmission energy of 3 eV, as determined by measuring

the photoelectron spectra of Ar, O_2, and N_2 by He(I) radiation with the procedure described elsewhere.[15,18] Spurious effects due to the geomagnetic field have been reduced to ≤ 20 mG by a μ-metal shielding.

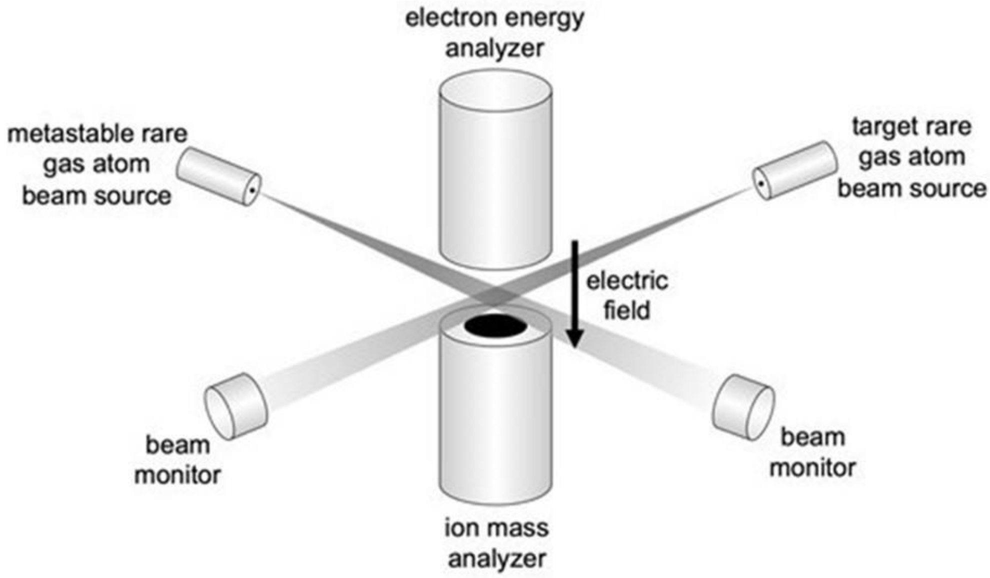

Supplementary Fig. 1

The schematic view of the apparatus used for Ne^*-Ar, N_2 and NH_3 chemi-ionization studies located at University of Perugia.

Supplementary References

[1] P. E. Siska, *Rev. Mod. Phys.***1993**, *65*, 337-412.

[2] B. Brunetti and F. Vecchiocattivi, *Current Topic on Ion Chemistry and Physics*, Ng, C. Y., Baer, T., Powis I., Eds.; John Wiley &Sons Ltd: New York, 1993; pp 359-445.

[3] W. H. Miller and H.; Morgner, *J. Chem. Phys.* **1977**, *67*, 4923-4930.

[4] S. Falcinelli, F. Vecchiocattivi, F. Pirani, *Phys. Rev. Lett.* **2018**, *121*, 163403.

[5] B. G. Brunetti, P. Candori, S. Falcinelli, F. Pirani, F. Vecchiocattivi, *J. Chem. Phys.* **2013,** *139***,** 164305.

[6] F. Pirani, S. Brizi, L. F. Roncaratti, P. Casavecchia, D. Cappelletti, F. Vecchiocattivi, *Phys. Chem. Chem. Phys.* **2008**, *10*, 5489-5503.

[7] S. Falcinelli, F. Vecchiocattivi, F. Pirani, *Commun. Chem.* **2020**, *3(1)*, 64.

[8] V. Aquilanti, R. Candori, F. Pirani, *J. Chem. Phys.* **1988**, *89*, 6157-6164.

[9] E. E. Nikitin and R. N. Zare, *Mol. Phys.* **1994**, 82, 85-100.

[10] F. Pirani, G. S. Maciel, D. Cappelletti, V. Aquilanti, *Int. Rev. Phys. Chem.* **2006**, *25*, 165-199.

[11] H. Hotop, A. Niehaus, *Chem. Phys. Lett.* **1971**, *8*, 497-500.

[12] V. Hoffmann, H. Morgner, *J. Phys. B: Atom. Molec. Phys.* **1979**, *12*, 2857-2874.

[13] K. Ohno, H. Mutoh, Y. Harada, *J. Am. Chem. Soc.* **1983**, *105*, 4555-4561.

[14] K. Ohno, *Bull. Chem. Soc. Japan* **2004**, 77, 887-908.

[15] B. G. Brunetti, P. Candori, D. Cappelletti, S. Falcinelli, F. Pirani, D. Stranges, F. Vecchiocattivi, *Chem. Phys. Lett.* **2012**, *539-540*, 19-23.

[16] S. D. S. Gordon, J. Zou, S. Tanteri, J. Jankunas, A. Osterwalder, *Phys. Rev. Lett.* **2017**, *119***,** 053001.

[17] S. D. S. Gordon, J. J. Omiste, J. Zou, S. Tanteri, P. Brumer, A. Osterwalder, *Nat. Chem.* **2018**, *10*, 1190-1195.

[18] H. Hotop, *J. Electron. Spectrosc. Relat. Phenom.* **1981**, *23*, 347-365.

[19] B. A. Jacobs, W. A. Rice, P. E. Siska, *J. Chem. Phys*. **2003**, *118***,** 3124-3130.
